# Depletion of Ku70/80 reduces the levels of extrachromosomal telomeric circles and inhibits proliferation of ALT cells

**DOI:** 10.18632/aging.100308

**Published:** 2011-04-15

**Authors:** Baomin Li, Sita Reddy, Lucio Comai

**Affiliations:** ^1^ Department of Molecular Microbiology and Immunology, Institute for Genetic Medicine, Keck School of Medicine, University of Southern California, Los Angeles, CA 90033, USA; ^2^ Department of Biochemistry and Molecular Biology, Institute for Genetic Medicine, Keck School of Medicine, University of Southern California, Los Angeles, CA 90033, USA

**Keywords:** Ku, telomeres, t-circles, ALT, cancer, aging

## Abstract

In normal cells, telomeres shorten each time a cell divides ultimately resulting in cell senescence. t In contrast, cancer cells counteract the loss of telomeric DNA either by inducing the expression of telomerase or by activating the Alternative Lengthening of Telomeres (ALT) pathway. ALT cells are characterized by heterogeneous telomeres and the presence of extrachromosomal circular double-stranded DNA molecules containing telomeric repeat sequences. These telomeric circles (t-circles) are thought to be generated through a recombination process and utilized as templates for telomere elongation by rolling-circle-replication, although their precise mechanism of formation and role in telomere maintenance and cell proliferation is largely unknown. Here we show that shRNA-mediated knockdown of the Ku70/80 heterodimer, a factor with functions at both pathological and natural DNA ends, inhibits ALT cell growth and results in a significant decrease in the levels of t-circles without affecting overall telomere length. These findings demonstrate that non homology-based processes contribute to the maintenance of t-circles and proliferation of ALT cells.

## INTRODUCTION

ALT is a mechanism of telomere maintenance that is utilized by a portion of cancers, particularly in tissues of mesenchymal origin. Telomeres in ALT cells are heterogeneous in length due to rapid deletions and elongations, which are thought to occur through high rates of interchromosomal recombination including a process termed telomere sister chromatid exchange (T-SCE). Significantly, there is no increase in rates of general homologous recombination in these cells, suggesting that the mechanism of hyper-recombination is telomere-specific. ALT cells are also characterized by the presence of extrachromosomal linear and circular telomere DNA molecules [1]. The extrachromosomal t-circles can in principle be utilized as templates for telomere elongation by rolling-circle-replication and therefore play an important role in the process of telomere maintenance in the absence of telomerase [1]. t-circles formation in ALT cells occurs through a process that requires the recombination proteins X-ray repair cross-complementing 3 (XRCC3) and Nijmegen breakage syndrome 1 (NBS1), as down-regulation of either one of these factors causes a dramatic decrease in the levels of t-circles in these cells [2]. However, MUS81, an endonuclease implicated in the regulation of telomere recombination, does not influence t-circle formation in ALT cells [3], suggesting that the precise contribution of the recombination machinery and the possible involvement of other pathways to the formation and maintenance of t-circles in human ALT cells remain to be defined.

Ku70/80 is an heterodimer that was first identified as a component of non-homologous end joining (NHEJ), an error-prone pathway involved in the repair of DNA double-strand breaks. More recently, Ku70/80 has been detected at telomeres and implicated in the regulation of telomere maintenance in a range of organisms including yeast, plants, mice and humans [4]. At first, since fusion of telomeres leads to the formation of dicentric chromosomes and chromosome breaks, it seemed paradoxical that Ku70/80, a protein that facilitates the joining of DNA ends, is localized at telomeres. Further analysis however suggested that a likely explanation for these observations is that Ku70/80 plays distinct roles at pathological and natural DNA ends. Indeed, Ku70/80 has been proposed to perform at least two functions at telomeres. First, it protects telomeres ends from degradation, and second it regulates telomere length, possibly through a functional interaction with the telomerase holoenzyme [5, 6]. However, how Ku70/80 operates at telomere seems to differ among species. For example, cells from Ku70 or Ku80 knockout plants show extreme telomere elongation while yeast lacking Ku70/80 display loss of telomeric repeats [7, 8]. Moreover, Ku70/80 has been shown to contribute both positively and negatively to telomere length homeostasis in mice [9-11]. While further studies are needed to reconcile these observations, it is likely that the differences in telomere length homeostasis induced by depletion of Ku70/80 in distinct systems reflect natural species-specific variations in Ku function and telomere biology. Interestingly, recent studies have demonstrated that inactivation of Ku70/80 induces the formation of t-circles in plant cells [12] and in human telomerase-positive cancer cells [13]. However, t-circle formation in Ku-deficient plants is not suppressed by inactivation of genes involved in the homologous recombination pathway, including XRCC3 [12]. A key molecular partner of Ku70/80, the Werner syndrome protein (WRN), is also involved in t-circle formation [14]. Interestingly, as observed in Ku70 or Ku80-deficient plants, t-circles in telomerase-positive human fibroblasts lacking WRN are refractory to XRCC3 depletion. Yet, WRN is required for the formation of t-circles in cells expressing TRF2^DB^ [14], a mutant form of the telomere repeat binding factor TRF2 which induces rapid telomere deletions and t-circles formation in normal fibroblast [14]. Collectively, these results suggest that in a context-dependent manner, distinct proteins can differentially influence the formation of t-circles. Here we investigate the role of Ku70/80 in ALT cells and demonstrate that down-regulation of this factor inhibits cell proliferation and significantly reduces the levels of t-circles without affecting telomere length or overhang signal. Thus, Ku70/80 is an essential factor for ALT cell survival and its function is required for the maintenance of extrachromosomal t-circles in these cells.

## RESULTS

**Figure 1.**
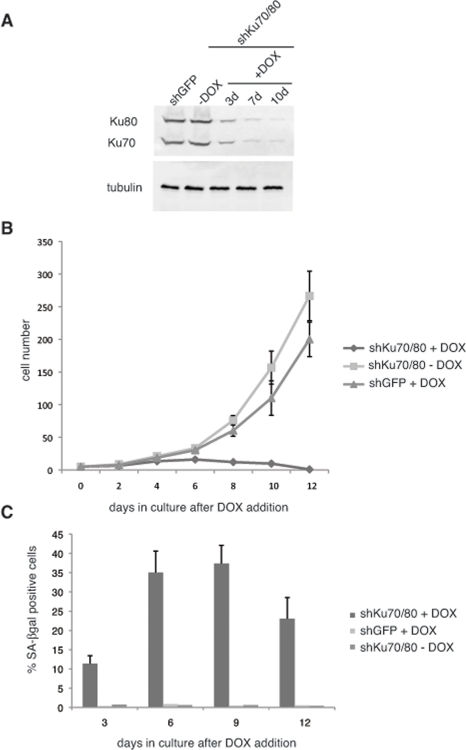
Short hairpin RNAs (shRNAs) for Ku70/80 inhibit the proliferation of ALT cells (**A**) shRNAs for Ku70/80 reduce the levels of Ku70/80 in CCL75.1 cells. CCL75.1 cells were infected with lentiviruses for the conditional expression of shRNAs targeting Ku70 and Ku80 or GFP and cultured for 3, 7 and10 days in the presence of 1.0 mg/ml Doxycycline (DOX). The levels of Ku70 and Ku80 were analyzed by immunoblotting with antibodies against Ku70 and Ku80. Antibodies against tubulin were used as a loading control. We estimated that 7 to 10 days after induction, Ku70/80 shRNAs-expressing cells display an 80 to 85% decrease in Ku70/80 expression compared to GFP shRNAs control. (**B**) Growth curves of CCL75.1 expressing shRNAs for Ku70/80 or GFP. After 5 days in Geneticin and hygromycin selection, 1.0ug/ml DOX was added to the media and the growth rate of each CCL75.1 cell line was measured by counting viable cells every 2 days. Cells were seeded at a low density, and the medium was changed every 2 days. Values represent the mean ± the standard deviation of three experiments (*n* = 3). (**C**) Detection of SA-β-gal activity. CCL75.1 cells transduced with lentiviruses for the conditional expression of shRNAs targeting K70/80 or GFP were grown for 3, 6, 9, or 12 days after addition of DOX and were stained for SA-βgal activity as previously described [14]. Values are the mean ± the standard deviation of three independent experiments (*n* = 3) carried out in duplicates in which 500 cells were scored for SA-β galactosidase. Student's *t* test was used to evaluate differences in means between two groups, and *P* < 0.05 was considered statistically significant. ALT cells (CCL75.1 and Saos2) were purchased from ATCC.

We set out to determine the effect of downregulation of Ku70/80 on ALT cell proliferation. For this purpose, we transduced CCL75.1 ALT cells with lentiviral vectors for the conditional expression of shRNAs targeting human Ku70 and Ku80. We reasoned that down regulation of both members of the heterodimer should prevent possible effects caused by the presence of free subunits. Transduced cells were subjected to Geneticin and hygromycin selections and the kinetics of Ku70/80 downregulation was monitored for 10 days following doxycycline (DOX) addition to the cell culture. As shown in Figure 1, within three days of induction, Ku70 and Ku80 levels decline more than 65% and then levels off to approximately 14% compared to control cells expressing shRNAs targeting an unrelated factor (GFP). Within this time frame, ALT cells transduced with the control silencing vector do not show any detectable difference in growth rate nor display morphological changes when compared to ALT cells culture in the absence of DOX (Figure 1b). In contrast, ALT cells expressing Ku70/80 shRNAs grow poorly when compared to either the same cells cultured in the absence of DOX or ALT cells expressing shRNAs for GFP treated with DOX, and the culture undergo proliferative arrest within 12 days after induction. During this time period, a significant number of cells display replicative senescence-like phenotype, exhibiting a flattened morphology, and between 25 to 35% of the cells stained positive for the senescence-associated β-galactosidase (SA-βgal) activity (Figure 1c). The percentage of senescent cells does not increase with passage but, rather, display a small decline, and the remaining cells eventually succumb to cell death (data not shown). The growth defects triggered by depletion of Ku70/80 in ALT cells resembles that caused by downregulation of the MRE11/RAD50/NBS1 (MRN) complex using shRNAs targeting MRE11 and NBS1 (Supplementary Figure S1). The MRN complex has previously been shown to play a role in the maintenance of telomeres in these cells, although knockdown of individual subunits of this complex results only in a slight decrease in proliferation [2, 15].

**Figure 2.**
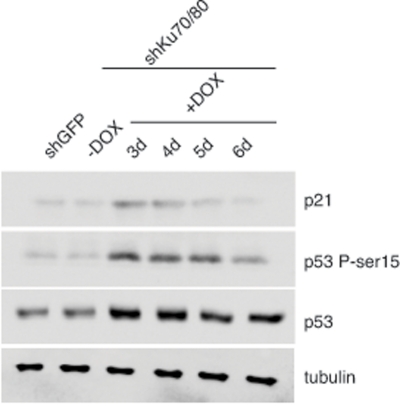
Depletion of Ku70/80 in CCL75.1 cells induces transient p53 activation The levels of p21, p53, and p53 phosphorylated at serine 15 were assessed by Western blotting in extracts prepared from CCL75.1 cells collected either before, or 3 to 6 days after addition of DOX. Tubulin serves as a loading control. Western blot analyses were performed with antibodies against p53, p53-phosphoserine15, p21, and tubulin.

To determine whether the reduced cell proliferation is linked to cellular stress, we examined the levels of p53 and its downstream target p21 in cells lysates prepared from ALT cells transduced with lentiviruses for the conditional expression of shRNAs for Ku70/80 or GFP at 3 to 6 days after induction with DOX. Western blot analysis shows an increase in the levels of p53 in ALT cells depleted of Ku70/80 compared to controls (Figure 2). Moreover, the Ku70/80-depleted cells display a transient increase in the levels of p21 and phospho-rylation of p53 at serine 15, an hallmark of DNA damage-induced p53 activation. In contrast, the levels of p53, p21, and Ser15 phosphorylation do not change after expression of shRNAs targeting GFP. The cellular response to down regulation of Ku70/80 closely resembles that of ALT cells depleted of the MRE11/RAD50/NBS1 complex, as shown by analysis of ALT cells transduced with lentiviral vectors for the expression of shRNAs targeting MRE11/NBS1 (Supplementary Figure S2).

**Figure 3.**
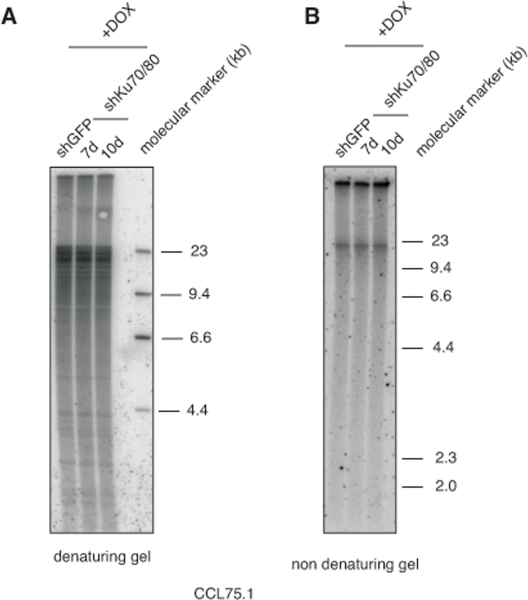
Depletion of Ku70/80 in CCL75.1 cells does not affect telomere length nor overhangs signal (**A**) Terminal Restriction Fragment (TRF) analysis. CCL75.1 cells transduced with lentiviruses for the conditional expression of shRNAs targeting Ku70 and Ku80 or GFP were harvested at 7 (shKu70/80) and 10 days (shKu70/80 and shGFP) after induction with DOX. Equal amounts of genomic DNA digested with HinfI and RsaI were separated by electrophoresis on a 0.8% agarose gel and analyzed by Southern blotting with a radiolabeled (CCCTAA)_4_probe. The molecular mass standards shown on the right lane were generated by digestion of lambda DNA with the restriction endonuclease HindIII. (**B**) Telomere 3′ overhang signal analysis. CCL75.1 cells transduced with lentiviruses for the conditional expression of shRNAs targeting either Ku70/80 or GFP were harvested at 7 (shKu70/80) and 10 days (shKu70/80 and shGFP) after induction with DOX. Equal amounts of genomic DNA digested with HinfI and RsaI were separated by electrophoresis on a 0.8% agarose gel. The gel was dried under native conditions and then hybridized to a radiolabeled (CCCTAA)_4_probe.

**Figure 4.**
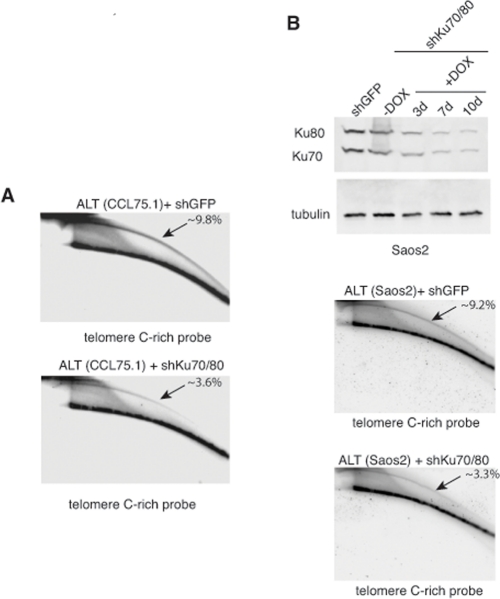
Depletion of Ku70/80 reduces the levels of t-circles in CCL75.1 and Saos2 cells (**A**) Genomic DNA isolated from CCL75.1 cells expressing shRNAs targeting Ku70/80 or GFP for 7 days was digested with HinfI and RsaI, separated by 2DGE, blotted, and probed with a telomeric (CCCTAA)_4_probe. The samples shown in each panel were run and processed in parallel under the same hybridization and washing conditions. The approximate levels of t-circles present in each sample (expressed as a percentage of the total telomeric DNA) were estimated as in [14] and is shown in the upper right corner. (**B**) (top panel) Saos2 cells were infected with lentiviruses for the conditional expression of shRNAs for Ku70and Ku80 or GFP and analyzed either before or 3, 7 or 10 days after the addition of 1.0 mg/ml DOX to the media. The levels of Ku70 and Ku80 were determined by Western blotting with antibodies against Ku70 and Ku80. We estimated that Ku70/80 shRNAs reduce Ku70/80 expression ~80% compared to GFP shRNAs control. Antibody against tubulin was used as a loading control. (middle and bottom panels) DNA isolated from Saos2 cells 7 days after DOX addition was digested with HinfI and RsaI, separated by 2DGE, blotted, and probed with a telomeric (CCCTAA)_4_probe. The samples shown in each panel were run and processed in parallel under the same hybridization and washing conditions and the approximate levels of t-circles present in each sample (expressed as a percentage of the total telomeric DNA) is shown in the upper right corner.

**Figure 5.**
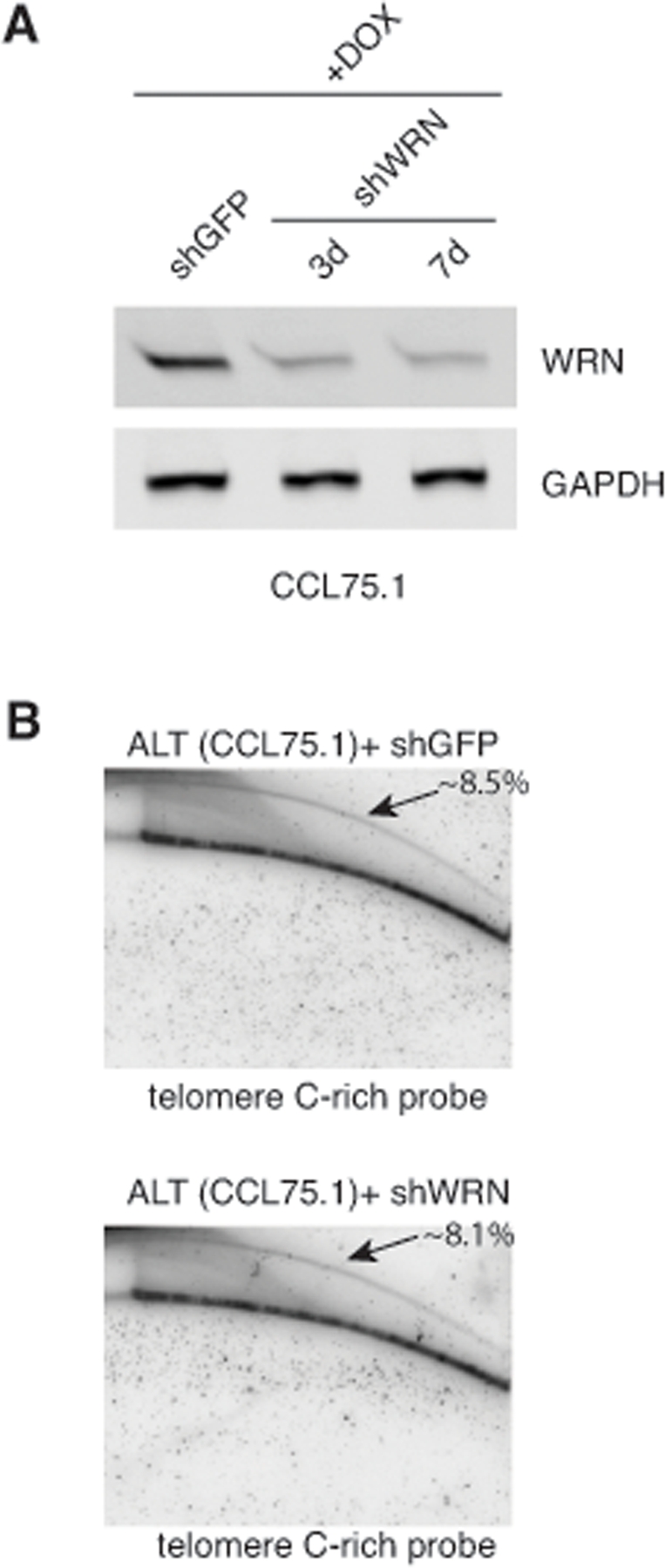
Depletion of WRN does not influence the levels of t-circles in CCL75.1 cells (**A**) CCL75.1 cells were infected with lentiviruses for the conditional expression of shRNAs for WRN or GFP and analyzed 3 days (shWRN) and 7 days (shWRN and shGFP) after the addition of 1.0 mg/ml DOX to the media. Protein levels were determined by Western blotting with WRN antibodies. We estimated that WRN shRNAs reduce its cognate gene expression ~75% compared to GFP shRNAs control after 7 days of DOX induction. Antibody against GAPDH was used as a loading control. (**B**) Genomic DNA isolated from CCL75.1 cells expressing shRNAs targeting WRN or GFP for 7 days was digested with the restriction endonucleases HinfI and RsaI, separated by 2DGE, blotted, and probed with a telomeric (CCCTAA)_4_ probe. The samples shown in each panel were run and processed in parallel under the same hybridization and washing conditions. The approximate levels of t-circles present in each sample (expressed as a percentage of the total telomeric DNA) were estimated as in [14] and is shown in the upper right corner.

Genetic knock-out of the Ku80 gene in telomerase-positive human cells results in reduced cell proliferation and is accompanied by dramatic shortening and loss of telomeres [16, 17]. Moreover, 50% reduction in the expression of Ku80 either by small interfering RNA (siRNA) or by functional inactivation of one allele in two telomerase-positive carcinoma cell lines, HeLa and HCT116, is sufficient to alter telomere homeostasis, as demonstrated by rapid telomere shortening and, at least in one of the studies, stronger telomere overhang signal [16, 18]. To determine whether depletion of Ku70/80 influences telomere homeostasis in ALT cells, we measured telomere length, single-stranded telomere overhang signal and t-circles in ALT cells transduced with lentiviruses for the conditional expression of shRNAs targeting Ku70 and Ku80. Cells were subjected to selections and DNA was isolated before induction and at 7 and 10 days after DOX addition. DNA was digested with HinfI and RsaI, resolved on agarose gel and hybridized to a radiolabeled telomeric probe under either denaturing or native conditions. This analysis show that within the time frame of this experiment, depletion of Ku70/80 does not significantly influence overall telomere length or the single-stranded telomeric overhang (Figure 3). In parallel, DNA isolated from the transduced cells was separated by 2-dimensional gel electrophoresis (2-DGE) and probed with a radiolabeled telomeric probe. This analysis shows that depletion of Ku70/80 is accompanied by a significant decrease in the intensity of the arc representing extrachromosomal t-circles (Figure 4). To corroborate this finding, we analyzed telomere length, overhang signal and t-circles in another ALT cells line, Saos2, and show that, as observed in CCL75.1, depletion of Ku70/80 in Saos2 does not affect overall telomere length nor 3′ overhang but results in a significant decrease in the levels of t-circles (Figure 4 and Supplementary Figure S3). These results indicate that Ku70/80 downregulation negatively influences t-circle formation in ALT cells, thus implicating non-homology-based processes in t-circles homeostasis in these cells. In contrast, shRNA-mediated silencing of the Werner syndrome protein (WRN), a factor that binds to Ku70/80 and has been implicated in the repression of t-circles in telomerase-positive cells [14], does not alter the levels of t-circles in ALT cells (Figure 5). As a control we show that downregulation of MRE11/NBS1 in both CCL75.1 and Saos2 cells also results in a significant reduction in the levels of t-circles (Supplementary Figure S4), which is in agreement with published results [2].

**Figure 6.**
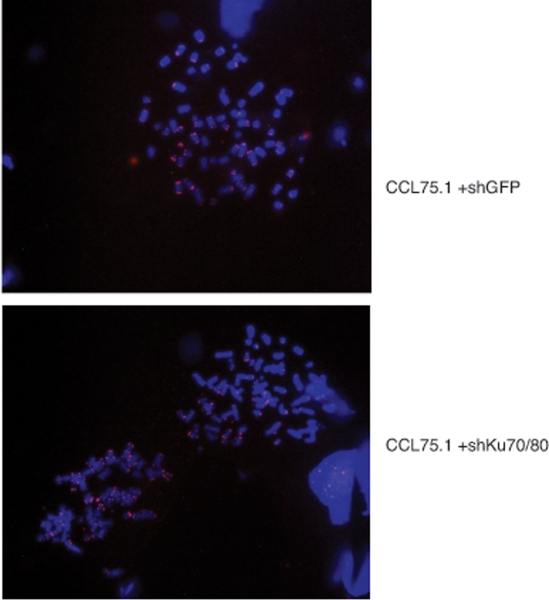
Telomere FISH analysis of ALT cells expressing shRNAs for Ku70/80 or GFP CCL75.1 cells expressing shRNAs for Ku70/80 or GFP were treated with 0.5 mg/ml of colcemid for 1.0 hour, harvested by trypsinization and swollen in 0.075M KCl for 20 min. The cells were fixed and washed with methanol:acetic acid (3:1) for three times and dropped into slides. Images shown are representative of more than 50 metaphases analyzed for each cell line.

To further assess whether depletion of Ku70/80 in ALT cells induces gross chromosome alterations, we performed FISH analysis of metaphase spreads using a telomere-PNA probe. As documented before [19], ALT cells display chromosomes with various chromosomal abnormalities as well as a wide range of telomere signal intensities including telomere signal free ends (Figure 6). Analysis of the metaphase spreads from Ku-depleted ALT cells did not reveal any significant difference in the distribution of telomere signals nor an increase in the frequency of chromosome abnormalities compared to control ALT cells (Figure 6).

## DISCUSSION

Ku70/80 is an heterodimeric factor that plays a critical role in the repair of double strand breaks by non-homologous end joining (NHEJ). In this process, Ku is though to facilitate the ligation of broken DNA ends by promoting the recruitment of nuclease, ligases and polymerases necessary for the repair [20]. As DNA end joining would be detrimental to chromosome ends, it was though that Ku has to be kept away from telomeres to prevent deleterious telomere-telomere fusion. Indeed, the formation of telomere-telomere fusion under pathological conditions has been shown to require DNA ligase IV, the ligase required for classical NHEJ [21]. It was therefore unexpected to find that Ku70/80 plays a vital role in controlling telomere length homeostasis, from yeast to humans. However, lack of Ku70/80 function differentially affects cells from distinct species, and in human telomerase-positive cancer cells, Ku70/80 has been shown to perform a protective function, as its depletion results in rapid telomere shortening, chromosome fusion and gross chromosomal rearrangements [16, 18]. The mechanism by which Ku70/80 protects telomeres is yet to be determined, but may involve, at least in part, physical and functional interactions with telomerase, components of the shelterin complex, or other telomere-associated factors [14, 22, 23]. Interestingly, the formation of telomere fusion in these cells implicates an alternative end-joining mechanism independent of Ku70/80 [24]. In this study we examined whether Ku is involved in telomere homeostasis in ALT cells, a type of human cancer cells that utilize a telomerase-independent mechanism of telomere maintenance, and define its role in the proliferation of these cells. Telomeres in ALT cells are highly heterogeneous in length and are maintained through a mechanism involving recombination [25-27], although the exact mechanism of this pathway remains to be elucidated. ALT cells produce extrachromosomal t-circles, possible products of intratelomeric recombination and t-loop resolution [28, 29]. Our study demonstrates that Ku depletion inhibits ALT cell proliferation and reinforces the concept that Ku70/80 is an essential factor in human cells. Depletion of Ku70/80 results in the activation of the p53 pathway within three days after shRNAs induction, as demonstrated by phosphorylation of p53 at ser15 and upregulation of p21. However, this activation appears to be transient, as the levels of p21 and phosphoserine-15 p53 rapidly decline and reach basal levels after 5 days. This result suggests that that activation of the p53 pathway is unlikely to be the signal triggering the decline in cell proliferation. Consistent with this interpretation, depletion of Ku70/80 causes a similar decline in the proliferation of Saos2, ALT cell line which lacks functional p53 (data not shown). In contrast, we noted a correlation between activation of the p53 pathway and the transient increase in the percentage of senescent cells, possibly suggesting that these two processes are functionally linked.

Although the growth rate of ALT cells depleted of Ku70/80 is severely limited, these cells do not show the massive telomere deletions observed in telomerase-positive human cells lacking Ku70/80, suggesting that telomere shortening is not responsible for the growth arrest of ALT cells. This conclusion is supported by FISH analysis of metaphase spreads, which shows that Ku-depleted ALT cells do not display any significant difference in the overall distribution of telomere signals and frequency of telomere signal-free ends nor increased chromosomal instability compared to control ALT cells. In contrast, ALT cells depleted of Ku70/80 display a marked decrease in the levels of t-circles, a prominent feature of ALT cells that has been implicated in telomere maintenance. The correlation between altered t-circle homeostasis and the reduced proliferative capacity of these cells is intriguing, although any functional relationship between these two phenotypes remains to be established. The decline in the levels of t-circles in ALT cells by depletion of Ku70/80 was unexpected, as loss of Ku80 in telomerase-positive human cells results in the accumulation of t-circles [13]. An explanation for these seemingly paradoxical results is that Ku70/80 function at telomeres is likely influenced by the structure and organization of telomeres and possibly, telomerase itself. The decline in the levels of t-circles caused by depletion of Ku70/80 closely resembles that shown by us and others in ALT cells depleted of MRE11/NBS1, suggesting that both complexes participate in the process that generate and maintain these extrachromosomal molecules in these cells. In contrast, depletion of WRN does not result in altered t-circle formation, indicating that this factor is not required in this process in cells that use alternative lengthening of telomeres. Indeed, lack of WRN seems to favor an ALT-like phenotype, as there is evidence that immortalized cell lines lacking WRN maintain telomere length in the absence of telomerase [30] and WRN-deficient, telomerase-negative mouse cells display features of ALT [31]. Yet, lack of WRN stimulates the formation of t-circles in telomerase-positive cells, suggesting that distinct mechanisms are involved in the formation of t-circles in cells that maintain telomere length by ALT or telomerase. Moreover, as alternative lengthening of telomeres remains a poorly defined process, we cannot rule out that distinct mechanisms of ALT also exists.

## MATERIALS AND METHODS

### Cell lines and antibodies

ALT cells (CCL75.1 and SAOS2) were purchased from ATCC. WS fibroblast was immortalized by expression of hTERT (TERT catalytic subunit) as previously described [14]. ALT cells were cultured in Dulbecco modified essential medium (DMEM) supplemented with 10% fetal calf serum and 1% penicillin-streptomycin and maintained at 37°C in a humidified incubator at 5% CO_2_ and atmospheric oxygen.

Western blot analyses were performed with antibodies against Ku70 (sc-1486; Santa Cruz Biotechnology, Inc.), Ku80 (sc-1484 Santa Cruz Biotechnology, Inc.), MRE11 (polyclonal sc-5859; Santa Cruz Biotechnology Inc.), NBS1 (polyclonal sc-8580; Santa Cruz Biotechnology Inc.), p53 (monoclonal sc-100; Santa Cruz Biotechnology Inc.), p53-phosphoserine15 (monoclonal 9284S; Cell Signaling), p21 (polyclonal sc-397; Santa Cruz Biotechnology Inc.), tubulin (monoclonal SC-5286; Santa Cruz Biotechnology Inc.), and GAPDH (monoclonal SC-137179; Santa Cruz Biotechnology Inc.). Anti-rabbit, anti-goat, and anti-mouse immunoglobulin G horseradish peroxidase-coupled antibodies were purchased from Promega and Santa Cruz Biotechnology Inc.

### Construction of inducible lentiviral shRNA vectors

To generate lentiviral vectors for the conditional expression of shRNAs targeting Ku70, Ku80, WRN, NBS1, MRE11 and GFP, complementary oligonucleotides were annealed and cloned into a pENTT-miRc2 vector [32]. Target sequences are as follows: Ku 70: 5′-GAAGAGAACCTTGAAGCAAGT -3′; Ku80: 5′-GAGAACAAGGATGAGATTGCT-3′; WRN-a: 5′ TGAAGAGCAAGTTACTTGCCT-3′; WRN-b: ACAGGTGAACTTAGGAAACTT-3′ GFP-a: 5′-CGCAAGTCGACCCTGAGTTCA-3′; GFP-b: 5′-GTTCATCTGCACCACCGGCTT-3′; NBS1: 5′-GGAGGAAAGATGRCAATGTTAG-3′; MRE11: 5′-GATGAGAACTCTTGGTTTAAC-3′. The shRNAs sequences for human Ku70, Ku80, WRN, MRE11, NBS1 and GFP are specific for their respective targets and have no significant homology to any known human gene. miR-shRNAs vector were then generated by *in vitro* recombination between pENTT-miRc2 and pSLIK-Neo or pSLIK-Hyg using the Gateway LR Clonase Enzyme Mix Kit (Invitrogen). Recombinant lentiviruses were produced as described in [32]. For lentiviral infection, ALT cells in culture were trypsinized, seeded onto 10-cm tissue culture dishes, and incubated for 24 h at 37°C. The viral supernatant was then added to cultures that were approximately 60 to 70% confluent and further incubated at 37°C. After 6 h, the viral supernatant was removed and the cells were washed twice with PBS and incubated in DMEM containing 10% fetal bovine serum and 1% penicillin-streptomycin at 37°C. Transduced cells with shRNA were selected in DMEM supplemented with 400 mg/ml Geneticin (G418) and 200 mg/ml hygromycin for 5 days. 1.0 mg/ml doxycycline (DOX) was added to the media to induce the expression of shRNAs. The levels of down regulation of the target proteins were estimated by Western blotting.

### Cell growth curves and senescence-associated β-galactosidase (SA-βgal) assay

ALT cells were infected with lentiviruses for the condtional expression of shRNAs and cultured for 5 days in DMEM containing 400 μg/ml Geneticin and 200 mg/ml Hygromycin. Cells were plated in duplicate in 35-mm tissue culture dishes, and DMEM with 1.0 mg/ml DOX was added to the culture and changed every 2 days. On the indicated days after transduction, cells were harvested and counted for the growth curve analyses. Transduced cells grown for 3, 6, 9, 12 days were stained for SA-βgal activity as previously described [14]. Student's *t* test was used to evaluate differences in means between two groups, and *P* < 0.05 was considered statistically significant.

### Neutral-neutral two-dimensional gel electrophoresis (2DGE)

Genomic DNA isolation and 2 dimensional gel electrophoresis (2DGE) was performed as described in [14] with the following modifications. Genomic DNA was digested with HinfI and RsaI, extracted with phenol-chloroform, and precipitated with ethanol. Ten micrograms of HinfI/RsaI-digested genomic DNA was separated on a 0.5% agarose gel in 1× Tris-borate-EDTA at 1 V/cm for 18 h at room temperature. The gel was stained in 1× Tris-borate-EDTA containing 0.3 μg/ml ethidium bromide for 30 min, and then the lanes were cut and placed at 90° to the direction of electrophoresis, and 1.0% agarose containing 0.3 μg/ml ethidium bromide was poured around the first-dimension lane. The second dimension was run at 4 V/cm for 4 h at room temperature. The DNA was transferred to Hybond membrane by Southern blotting and hybridized with a (CCCTAA)_4_ probe. After hybridization, excess probe was washed from the membrane and the pattern of hybridization was visualized on X-ray film by autoradiography and PhosphorImager (Molecular Dynamics, Inc.) scanning of the membrane. The percentage of t-circles was estimated as described in [14].

### Telomere length analysis

ALT cells trasduced with lentiviruses for the conditional expression of shRNAs for Ku70/80, NBS1/MRE11 or GFP were maintained in media containing 400 μg/ml Geneticin, 200 mg/ml Hygromycin, and collected 7 or 10 days after addition of 1.0 mg/ml DOX. Genomic DNA was isolated by standard protocols, and equal amounts of DNA were digested with HinfI and RsaI. Two micrograms of DNA was separated on 0.8% agarose gels, transferred to Hybond membrane, and hybridized with a radiolabeled (CCCTAA)_4_ probe. Blots were exposed on films or PhosphorImager screens, and telomeric repeat signals were quantitated with ImageQuant software (Molecular Dynamics, Inc.).

### Single-strand G-rich telomere length analysis

Genomic DNA was isolated by standard protocols, and equal amounts of DNA were digested with HinfI and RsaI. DNA (6 μg) was separated on 0.8% agarose gel. The gel was dried at 50°C for 3 h, washed with 2× SSC (1× SSC is 0.15 M NaCl plus 0.015 M sodium citrate), and probed with a (CCCTAA)_4_ probe.

### Telomere PNA-FISH assay

For chromosome spreads, CCL75.1 cells expressing shRNAs for Ku70/80 or GFP were treated with 0.5 mg/ml of colcemide for 1.0 hour, then harvested by trypsinization, and swollen in 0.075M KCl for 20min. The cells were fixed and washed with methanol:acetic acid (3:1) for three times. Metaphase chromosomes hybridization assays were carried out following the recommendation of the Telomere PNA FISH kit (Dako) supporting protocol. Chromosomes were counter-stained with mounting medium containining DAPI.

## SUPPLEMENTARY MATERIALS


