# Supplementary material for: Depletion of Ku70/80 reduces the levels of extrachromosomal telomeric circles and inhibits proliferation of ALT cells
Source: Aging (Albany NY). 2011 Apr 15;3(4):395–406. doi: 10.18632/aging.100308 (PMC3117455; doi:10.18632/aging.100308)
Supplement: Supplementary file 1 [file aging-03-395-s001.pdf]

25. Bryan TM, Englezou A, Gupta J, Bacchetti S, Reddel RR. Telomere elongation in immortal human cells without detectable telomerase activity. EMBO J, 1995; 14: 4240-4248.
26. Murnane JP, Sabatier L, Marder BA, Morgan WF. Telomere dynamics in an immortal human cell line. EMBO J, 1994; 13: 4953-4962.
27. Dunham MA, Neumann AA, Fasching CL, Reddel RR. Telomere maintenance by recombination in human cells. Nat Genet, 2000; 26: 447-450.
28. Tomaska L, Nosek J, Kramara J, Griffith JD. Telomeric circles: universal players in telomere maintenance? Nat Struct Mol Biol, 2009, 16: 1010-1015.
29. Cesare AJ, and Griffith JD. Telomeric DNA in ALT cells is characterized by free telomeric circles and heterogeneous t-loops. Mol Cell Biol, 2004; 24: 9948-9957.
30. Marciniak RA, Lombard DB, Johnson FB, Guarente LA. novel telomere structure in a human alternative lengthening of telomeres cell line. Cancer Res, 2005; 65: 2730-2737.
31. Laud PR, Multani AS, Bailey SM, Wu L, Ma J, Kingsley C, Lebel M, Pathak S, DePinho RA, Chang S. Elevated telomere-telomere recombination in WRN-deficient, telomere dysfunctional cells promotes escape from senescence and engagement of the ALT pathway. Genes Dev, 2005, 19: 2560-2570.
32. Shin KJ, Wall EA, Zavzavadjian JR, Santat LA, Liu J, Hwang JI, Rebres R, Roach T, Seaman W, Simon MI, Fraser ID. A single lentiviral vector platform for microRNA-based conditional RNA interference and coordinated transgene expression. Proc Natl Acad Sci U S A, 2006; 103: 13759-13764.

SUPPLEMENTARY FIGURES

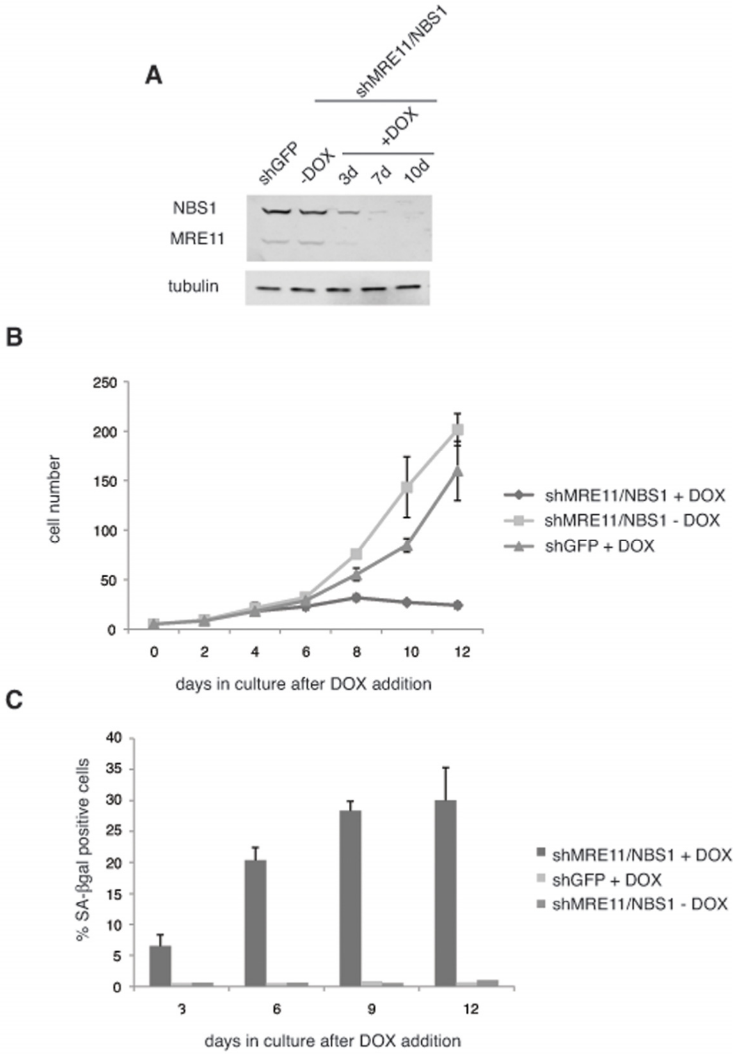

Supplementary Figure S1. Depletion of NBS1/MRE11 inhibits the growth of CCL75.1 cells. (**A**) shRNAs for NBS1 and MRE11 reduce the levels of NBS1 and MRE11 in ALT cells. CCL75.1 cells were infected with lentiviruses for the conditional expression of shRNAs for NBS1 and MRE11 or GFP and cultured for 3, 7 or 10 days in the presence of 1.0 mg/ml DOX. The level of NBS1 and MRE11 were analyzed by Western blotting with antibodies against NBS1 and MRE11 antibodies. Antibody against tubulin was used as a loading control. We estimated that shRNAs against MRE11/NBS1 reduce the levels of MRE11 and NBS1 more than 90% compared to shGFP control. (**B**) Cell growth analysis of CCL75.1 expressing shRNAs for NBS1 and MRE11. CCL75.1 cells infected with lentiviruses for the conditional expression of shRNAs against the indicated proteins were cultured for 5 days in media containing Geneticin and hygromycin and growth rates were measured by counting the cells every 2 days after the addition of 1.0 mg/ml DOX. Cells were seeded at a low density, and the medium was changed every 2 days. Values represent the mean ± the standard deviation of three experiments (*n* = 3). (**C**) Detection of SA-β-gal activity. CCL75.1 cells expressing shRNAs for NBS1/MRE11 or GFP were cultured for 12 days after DOX induction, fixed, and stained. The SA-βgal-positive cells among 500 cells were counted. Values are the mean ± the standard deviation of three independent experiments (*n* = 3) carried out in duplicate.

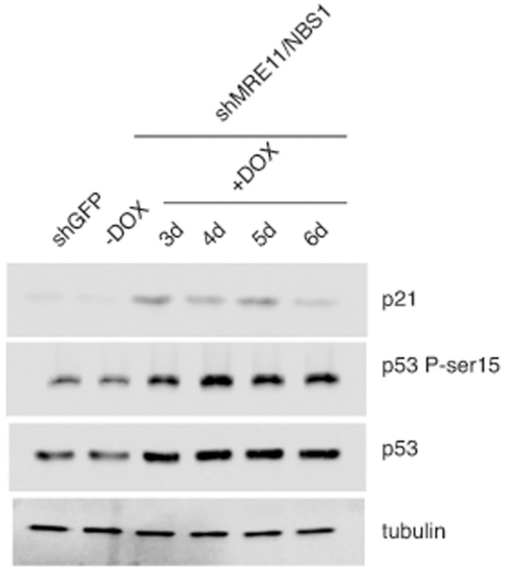

Supplementary Figure S2. Depletion of NBS1/ MRE11 induces a p53 response in CCL75.1 cells. The levels of p21, p53, and phosphorylated p53 on serine 15 in CCL75.1 cells were assessed by Western blotting of extracts prepared from cells collected before DOX induction, or at the indicated days after expression of shRNAs. Tubulin serves as a loading control.

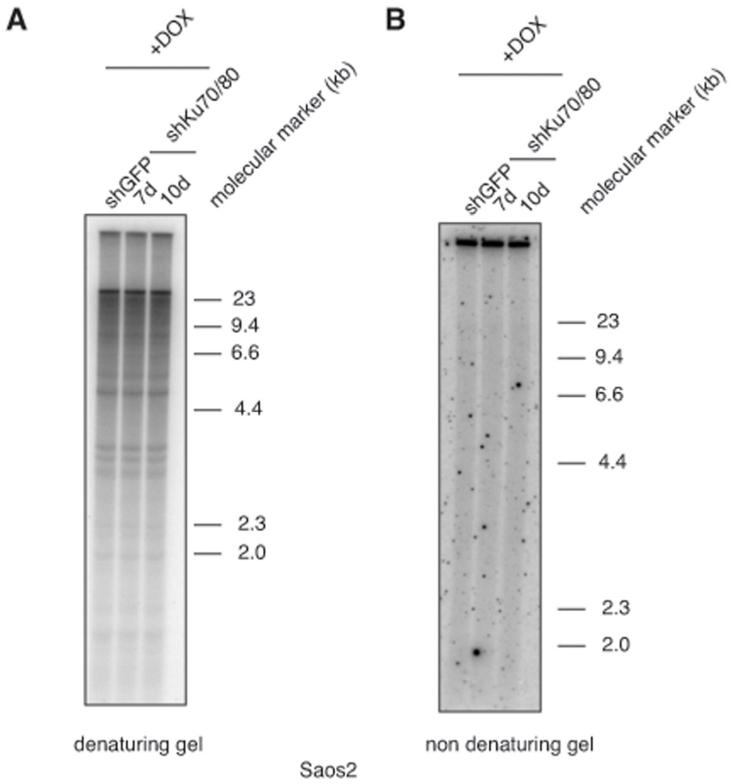

Supplementary Figure S3. Depletion of NBS1/MRE11 reduces the levels of t-circles in ALT cells. shRNAs for NBS/MRE11 reduce the level of NBS1/MRE11 in ALT cells. CCL75.1 cells (**A**) and Sos2 cells (**B**) were infected with lentiviruses for the conditional expression of shRNAs for NBS1 and MRE11 or GFP and cultured for 3, 7 or 10 days in the presence of 1.0 mg/ml DOX. The level of NBS1 and MRE11 were analyzed by Western blotting with antibodies against NBS1 and MRE11 (supplementary Figure S1 and (**B**)). Antibody against tubulin was used as a loading control. DNA, isolated from (a) CCL75.1 cells and (**B**) SAOS2 cells cultured for 7 days in the presence of DOX, was digested with HinfI and RsaI, separated by 2DGE, blotted, and probed with a telomeric $(CCCTAA)_4$ probe.

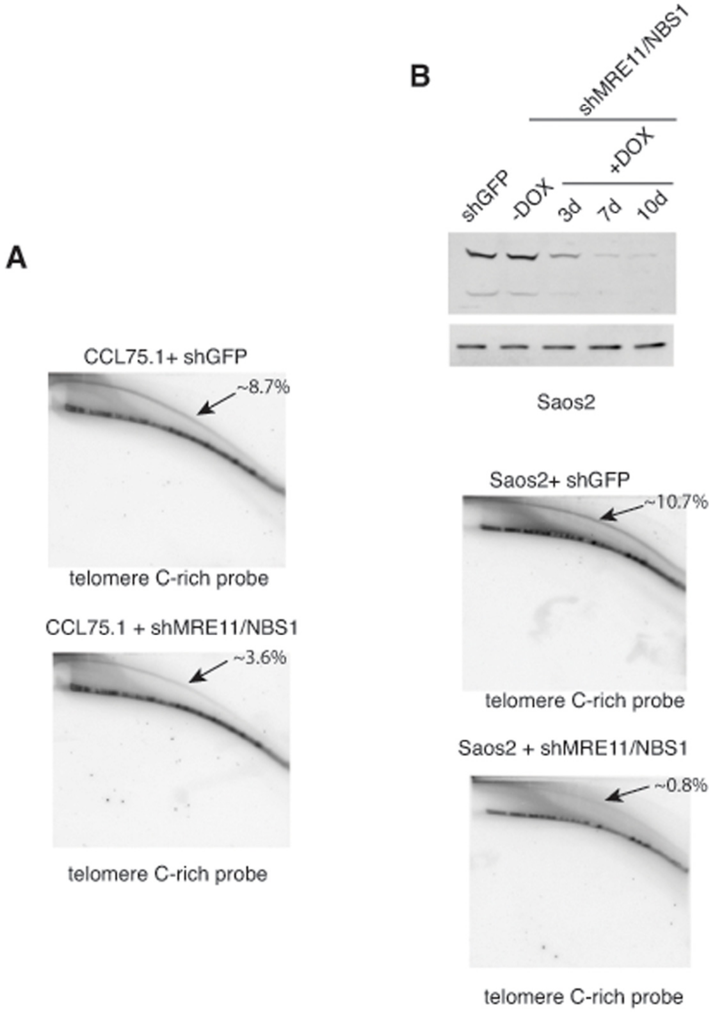

Supplementary Figure S4. Depletion of Ku70/80 does not influence telomere length in Saos2 cells. (**A**) Saos2 cells expressing shRNAs for Ku70/80 or GFP were harvested 7 or 10 days after the addition of DOX to the media. Equal amounts of genomic DNA digested with HinfI and RsaI were separated by electrophoresis on a 0.8% agarose gel and analyzed by Southern blotting with a radiolabeled $(CCCTAA)_4$ probe. (**B**) Soas2 cells expressing shRNAs for Ku70/80 or GFP were harvested 7 or 10 days after the addition of DOX to the media. Equal amounts of genomic DNA digested with Hinfl and RsaI were separated by electrophoresis on a 0.8% agarose gel, which was dried and then hybridized with a telomeric $(CCCTAA)_4$ probe under native conditions.
